# Incidence of penile cancer in Southeast Wales: A 15‐year retrospective review

**DOI:** 10.1002/bco2.70185

**Published:** 2026-08-13

**Authors:** Joseph Latham, Oliver Lane, David Gower, Adrian Sale, Anthony Shanahan, Mohammed Ali, Gareth Brown

**Affiliations:** ^1^ Cwm Taf Morgannwg University Health Board The Royal Glamorgan Hospital Llantrisant UK; ^2^ Cardiff and Vale University Health Board University Hospital Wales Cardiff UK

**Keywords:** epidemiology, incidence, penile cancer, penile intraepithelial neoplasia, public health, Wales

## Abstract

**Objectives:**

This study aims to determine the 15‐year incidence of penile cancer (PeCa) and penile intraepithelial neoplasia (PeIN) in Southeast Wales, evaluate regional variation and compare findings to UK and global rates.

**Materials and Methods:**

A retrospective review was conducted of all PeCa and PeIN cases diagnosed between January 2009 and December 2024 within the referral population served by the Royal Glamorgan Hospital, the tertiary centre for Southeast Wales. Postcodes were matched to local authorities and linked to mid‐year male population estimates from the Office for National Statistics. Crude incidence rates per 100 000 males were calculated for each region across the 15‐year study period.

**Results:**

A total of 298 cases were identified across 11 local authorities, representing 64% of the Welsh population. The mean crude incidence was 2.61 per 100 000 males (95% CI 1.59–3.62), ranging from 0.29 to 5.96. Blaenau Gwent demonstrated the highest incidence (5.96 per 100 000), followed by Merthyr Tydfil (3.68), Rhondda Cynon Taf (3.39), Newport (3.32) and Monmouthshire (2.90). Nine of 11 regions exceeded the reported UK average of 1.30 per 100 000, and 10 exceeded the global average of 0.80 per 100 000. Areas with higher incidence corresponded strongly with measures of socioeconomic deprivation.

**Conclusions:**

The incidence of PeCa and PeIN in Southeast Wales is substantially higher than previously reported UK and global rates. Regions with greater socioeconomic deprivation exhibit disproportionately elevated incidence. These findings highlight an urgent need for targeted public health interventions, including smoking cessation, HPV vaccination uptake and improved recognition of early penile lesions in primary care.

## 1 INTRODUCTION

Penile cancer (PeCa) is a rare malignancy. It is estimated to have a global incidence of 0.80 per 100 000 in 2018^1^ and a UK incidence of 1.30 per 100 000 as of 2009.^2^ Cancer Research UK reports 760 new cases of penile cancer per year, with this expected to rise to 1100 per year by 2038–2040. The 10‐year survival rate as of 2009–2013 is 68%; however, the vast majority of these deaths are preventable if detected early.^3^ The known risk factors for penile cancer include advancing age (the highest incidence being in men over 90 years),^3^ human papillomavirus (HPV) 6, 16 and 18,^4^ smoking (4.5‐fold increase),^4^ hygiene and phimosis (increased smegma known to be a carcinogen in animals).^1^ Other risk factors include ultraviolet A (UV‐A) exposure, penile trauma and socioeconomic deprivation.^4^ There is a strong socioeconomic correlation, and literature suggests that men in the most deprived quintile in England had a 52% higher incidence of PeCa compared with those in the least deprived quintile.^3^

PeCa incidence in Wales is poorly reported, with no publicly accessible data available at a regional level. The objective of this study is to explore and examine trends in PeCa and Penile Intraepithelial Neoplasia (PeIN) incidence in Southeast Wales from 2009 to 2024 and identify regional hotspots. By pinpointing areas with higher incidence, the study seeks to inform targeted public health strategies, including awareness campaigns and, where appropriate, local screening programs, to improve early detection, reduce mortality and enhance overall population health and disease prevention.

## 2 SUBJECTS/PATIENTS AND METHODS

Wales comprises 22 local authorities, of which 11 are in Southeast Wales, 5 in Southwest Wales and 6 in North Wales. Within this regional structure, the Royal Glamorgan Hospital serves as the designated tertiary referral centre for penile cancer across Southeast Wales. All PeCa and PeIN cases from this region are therefore treated by a single specialist service, enabling consistent case identification and facilitating comprehensive regional data collection. The 11 combined authorities of Southeast Wales—Blaenau Gwent, Bridgend, Caerphilly, Cardiff, Merthyr Tydfil, Monmouthshire, Newport, Powys, Rhondda Cynon Taf, Torfaen, Vale of Glamorgan—comprise two‐thirds (64%) of the Welsh population as of 2021.^5^ Retrospective data were collected from the Royal Glamorgan Hospital PeCa Multidisciplinary (MDT) meetings and operative records between 2009 and 2024. This dataset included patient postcode information for all confirmed cases of PeCa and PeIN, enabling analysis of geographical distribution and potential socioeconomic influences. Using the outward part of the patient's postcode (i.e., CF21 rather than CF21 0DC), these cases were assigned to their corresponding local authority before being aggregated to generate the total number of recorded PeCa/PeIN cases per region over the past 15 years. The incidence was calculated by using the total number of new cases of PeCa/PeIN over 15 years (per local authority) over the total 15‐year, mid‐year male population (per local authority) × 100 000. Mid‐year male population (per local authority) was obtained using the Office of National Statistics (ONS).^5^, ^6^

## 3 RESULTS

A total of 298 patients were included. The mean age at diagnosis was 64 years (median 67; range 19–95). Most patients were new diagnoses (*n* = 295), with recurrent disease in three patients (*n* = 3) who had prior disease before 2009 or relocated into the catchment area.

### 3.1 Diagnosis and histology

Of the 298 patients, PeIN was diagnosed in 87 patients (*n* = 87) and PeCa in 211 patients (*n* = 211). Histological subtypes among PeCa cases were squamous cell carcinoma (*n* = 206), melanoma (*n* = 2), sarcomatoid carcinoma (*n* = 2) and Kaposi sarcoma (*n* = 1).

Diagnosis was confirmed by direct biopsy in 291 patients (*n* = 291); however, the biopsy technique (punch vs. excisional) was not consistently recorded. In the remaining patients, diagnosis was based on radiological findings or was unspecified (*n* = 7).

### 3.2 Tumour grading (PeCa cohort)

Among the 211 PeCa cases, tumour grade was Grade 1 (*n* = 46), Grade 2 (*n* = 93), Grade 3 (*n* = 67) and Grade 4 (*n* = 3). Tumour grade was not recorded in two cases (*n* = 2).

### 3.3 Tumour staging (PeCa cohort)

Tumour stage was documented as T1 (*n* = 62), T1a (*n* = 21), T1b (*n* = 10), T2 (*n* = 59), T3 (*n* = 31) and T4 (*n* = 0). T stage was not recorded in 28 cases (*n* = 28). Nodal status was N0 (*n* = 172), N1 (*n* = 20), N2 (*n* = 10) and N3 (*n* = 4), with nodal status not recorded in five cases (*n* = 5). Metastatic status was M0 (*n* = 201) and M1 (*n* = 3), with M stage not recorded in seven cases (*n* = 7).

### 3.4 Initial treatments (PeCa and PeIN cohorts)

A total of 298 patients were included. The most frequently performed intervention was circumcision (*n* = 84), followed by glansectomy (*n* = 57), total penectomy (*n* = 47), excision (*n* = 36) and partial penectomy (*n* = 34). Less commonly utilised treatments included topical 5‐fluorouracil (*n* = 12), radiotherapy (*n* = 11) and glans resurfacing (*n* = 9). Radiotherapy was delivered due to patient choice (*n* = 7) or medical unfitness for surgery (*n* = 4). Palliative management (*n* = 3) and no recorded intervention (*n* = 3) were uncommon. Other treatments (*n* = 2) included laser therapy (*n* = 1) and one patient who moved away prior to definitive treatment (*n* = 1).

### 3.5 Additional treatments (PeCa cohort)

Lymph node surgery was common, with superficial inguinal lymph node dissection (SMILND) performed in 63 patients (*n* = 63), radical inguinal lymph node dissection (RILND) in 24 patients (*n* = 24) and pelvic lymph node dissection (PLND) in 11 patients (*n* = 11). Adjuvant or salvage oncological treatments included radiotherapy in five patients (*n* = 5) and chemotherapy in two patients (*n* = 2).

### 3.6 Local authority analysis

**FIGURE 1:**
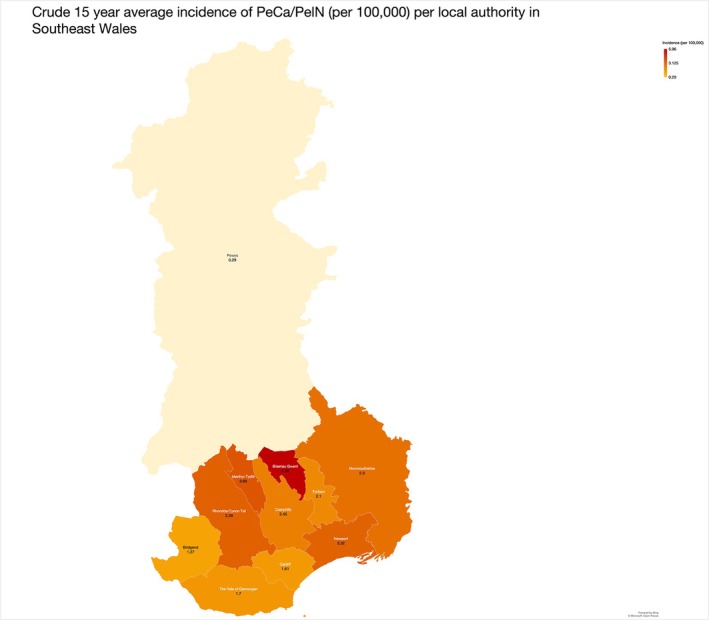
Crude 15‐year average incidence of PeCa/PeIN (per 100 000 males) per local authority in Southeast Wales.

There are 22 local authorities in the whole of Wales. This study included cases from the 11 authorities, representing the Southeast of Wales. All 11 local authorities reported at least one case of PeCa/PeIN. A total of 298 cases of PeCa/PeIN were identified across the 15‐year period between 2009 and 2024. The overall 15‐year average crude incidence was 2.61 per 100 000 males (95% CI: 1.59–3.62; range: 0.29–5.96). Blaenau Gwent local authority reported the highest incidence at 5.96 per 100 000, followed by Merthyr Tydfil, Rhondda Cynon Taf, Newport, Monmouthshire, Caerphilly, Torfaen, Vale of Glamorgan, Cardiff, Bridgend and Powys (3.68, 3.39, 3.32, 2.90, 2.45, 2.10, 1.70, 1.61, 1.27, 0.29 per 100 000, respectively), which are all (except Powys) above both the global incidence of 0.80 per 100 000 and all (except Powys and Bridgend) above the UK average of 1.30 per 100 000 (Table 1 and Figure 1).

**TABLE 1:** Penile cancer incidence, life expectancy, smoking‐related admissions and socioeconomic deprivation across Welsh local authorities.

Local authority	Number of cases over 15 years	Total male population over 15 years	Incidence of PeCa/PeIN (per 100 000 males)	Life expectancy (years)	Smoking‐attributed hospital admissions (per 100 000)	Socioeconomic deprivation measured in WIMD score (most derived 50% LSOAs in Wales)
Blaenau Gwent	32	536 898	5.96	76.0	1994	85
Bridgend	10	1 389 503	1.27	78.0	1519	56
Caerphilly	34	1 389 503	2.45	76.6	1772	63
Cardiff	45	2 792 917	1.61	78.3	1167	49
Merthyr Tydfil	17	461 577	3.68	75.4	1911	78
Monmouthshire	21	724 991	2.9	80.4	997	20
Newport	40	1 204 938	3.32	77.5	1375	60
Powys	3	1 051 326	0.29	79.5	1343	24
Rhondda Cynon Taf	63	1 856 247	3.39	77.1	1766	71
Torfaen	15	713 209	2.1	77.7	1462	57
Vale of Glamorgan	17	1 000 919	1.7	79.7	1222	35

### 3.7 Life expectancy and PeIN/PeCa incidence

**FIGURE 2:**
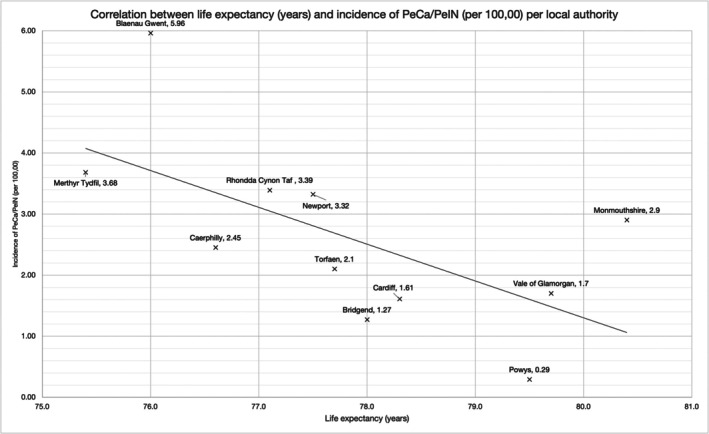
Correlation between life expectancy (years) and incidence of PeCa/PeIN (per 100 000 males) per local authority.

Local authority life expectancy (years) was obtained via the Office of National Statistics and compared with corresponding PeIN/PeCa incidence rates. Figure 2 demonstrates the correlation.

### 3.8 Smoking and PeIN/PeCa incidence

**FIGURE 3:**
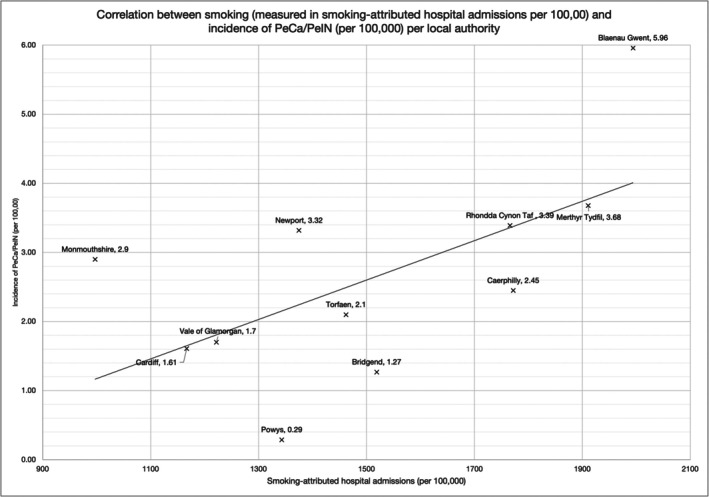
Correlation between smoking (measured in smoking‐attributed hospital admissions per 100 000 males) and incidence of PeCa/PeIN (per 100 000 males) per local authority.

Local authority smoking rates (measured in smoking‐attributed hospital admissions per 100 000) were obtained via the Office of National Statistics and compared with corresponding PeIN/PeCa incidence rates. Figure 3 demonstrates the correlation.

### 3.9 Socioeconomic deprivation and PeIN/PeCa incidence

**FIGURE 4:**
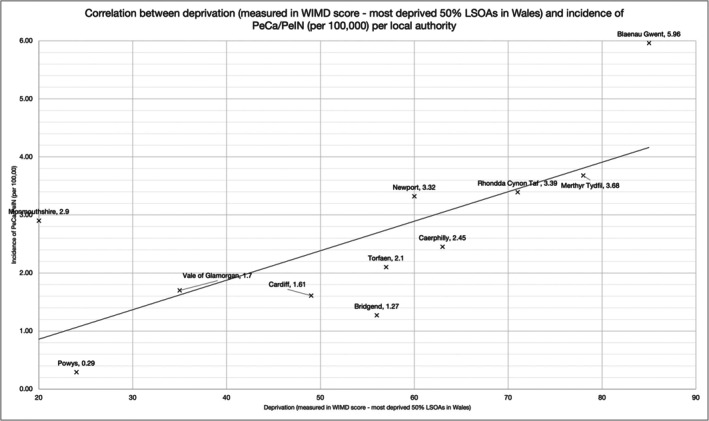
Correlation between socioeconomic deprivation (measured in WIMD score – most deprived 50% LSOAs in Wales) and incidence of PeCa/PeIN (per 100 000) per local authority.

Local authority socioeconomic deprivation (measured by the Welsh Index of Multiple Deprivation [WIMD]) was obtained from StatsWales and compared with corresponding PeIN/PeCa incidence rates. Figure 4 demonstrates the correlation.

## 4 DISCUSSION

Our study represents one of the largest regional analyses of PeCa/PeIN incidence in Wales, spanning over 15 years. However, it is a limited epidemiology study in that it only reports the crude, 15‐year average incidence rates. Nonetheless, it does provide meaningful and comparable data regionally, nationally and globally. The overall mean incidence of 2.61 per 100 000 males far exceeds currently reported UK averages (1.30 per 100 000)^2^ and global estimates (0.80 per 100 000).^1^

### 4.1 Possible explanations for the elevated incidence in Wales

Our data show that the incidence of PeCa/PeIN in Southeast Wales is 2.61 per 100 000—starkly higher than the UK average of 1.30 per 100 000.^2^ Several factors may explain this difference. First, this may represent a *true biological rise* in cases. Certain areas of Southeast Wales have a relatively older male population, higher smoking prevalence and greater levels of socioeconomic deprivation—all recognised risk factors for penile cancer.^4^, ^7^, ^8^, ^9^ These factors have been consistently associated with increased disease burden and poorer outcomes in population‐based studies.^7^, ^8^ However, the apparent rise could also reflect *improved case capture* rather than a genuine increase in incidence. The introduction of standardised cancer referral pathways, such as the NICE 2‐week‐wait (2WW) system and the NHS Faster Diagnosis Standard, has improved early identification and documentation of suspected cancers.^10^ Increased clinician awareness and public education may therefore have contributed to higher detection rates, similar to trends reported in England over the past two decades.^11^ It is important to note that there are no previously published Wales‐only incidence reports for comparison, as most national figures combine England and Wales. Given that the Royal Glamorgan Hospital is the single tertiary referral centre for PeCa/PeIN in Southeast Wales, our dataset likely provides a robust and accurate reflection of the true incidence in the region.

### 4.2 Regional analysis

When comparing regional variation between the local authorities, there appear to be trends in geographical incidence with certain ‘hotspot’ areas of exceptionally high incidence. To explain these fluctuations in local incidence, we must analyse the risk factors correlated with PeCa/PeIN. The most known risk factors are age, smoking, HPV infection and socioeconomic deprivation.^4^, ^7^, ^8^, ^9^

### 4.3 Life expectancy

There is a well‐documented link between increased age and increased risk of PeCa/PeIN.^9^ According to ONS 2023, the average life expectancy for males in the whole of Wales between 2021 and 2023 was 78 years of age. Within the Southeast Wales region, there is notable geographic variation in life expectancy, ranging from 75.4 years in Merthyr Tydfil to 80.4 years in Monmouthshire. Other local authorities fall within this spectrum, including Blaenau Gwent (76.0 years), Caerphilly (76.6 years), Rhondda Cynon Taf (77.1 years), Newport (77.5 years), Torfaen (77.7 years), Bridgend (78.0 years), Cardiff (78.3 years), Powys (79.5 years) and the Vale of Glamorgan (79.7 years)^12^ (Table 1). Although older age is considered a risk factor for PeCa/PeIN, regional life‐expectancy patterns in Southeast Wales suggest a reverse link—the lower the life expectancy, the higher the incidence PeCa/PeIN. For example, Merthyr Tydfil has the lowest life expectancy (75.4 years) and the second highest incidence of PeCa/PeIN (3.68 per 100 000). The next region with the lowest life expectancy is Blaenau Gwent (76.0 years), which has the highest incidence of PeCa/PeIN (5.96 per 100 000). This appears to be the general trend, and especially notable in Valley's region, where lower life expectancy seems to correlate with increased incidence of PeCa/PeIN. The only anomaly to this being Monmouthshire, where there is the highest life expectancy (80.4 years) but also the fifth highest incidence of PeCa/PeIN (2.90 per 100 000) (Figure 2). This highlights an important opportunity to recognise that areas of lower life expectancy interact with wider social determinants of health, and therefore targeted public health initiatives in these regions may help mitigate regional inequalities and support earlier diagnosis in higher risk communities.

### 4.4 Smoking

Smoking is a well‐established independent risk factor for penile cancer.^4^, ^7^ Public Health Wales data from 2013 to 2015^12^ indicate that the highest rates of smoking‐attributed hospital admissions occurred in Blaenau Gwent (1994 admissions per 100 000) and Merthyr Tydfil (1911 admission per 100 000) (Table 1). Notably, these regions correspond to the areas with the highest PeCa/PeIN incidence in Southeast Wales (Blaenau Gwent 5.96 and Merthyr Tydfil 3.68 per 100 000), as demonstrated in our study, reinforcing the link between tobacco exposure and disease (Figure 3). These findings stress the importance of improved smoking cessation strategies.

### 4.5 HPV

HPV is a recognised risk factor for PeCa and PeIN.^4^, ^7^, ^9^ There is a particularly strong association with HPV types 6, 16 and 18, which are detected in approximately 50% of PeCa and 80% of PeIN cases.^13^ This underscores the importance of HPV‐focused prevention measures.

Unfortunately, it is not possible to determine HPV prevalence by local authority, as such data is not available. Moreover, HPV infection is often asymptomatic, meaning that true population rates remain largely undetected. Consequently, the impact of this risk factor can only be interpreted indirectly: Areas with high rates of PeCa/PeIN may indicate populations with high rates of undetected HPV. Clustering of high‐incidence areas suggests a potential role for targeted HPV‐related measures within affected communities. Health prevention strategies could include strengthening efforts to promote and increase uptake of the HPV vaccine among boys aged 12–13, in line with the national vaccination programme,^14^ as well as exploring the feasibility of HPV screening to support earlier detection and reduce disease burden.

### 4.6 Socioeconomic deprivation

There is significant variation in socioeconomic deprivation throughout Southeast Wales. Welsh local authorities show a clear relationship between deprivation, measured by the Welsh Index of Multiple Deprivation (WIMD),^6^ and the incidence of PeCa/PeIN. The WIMD scores in the table reflect the average deprivation of the most deprived 50% of Lower‐layer Super Output Areas (LSOAs) within each authority, highlighting areas of greatest need. Authorities with higher deprivation scores, such as Blaenau Gwent (85), Merthyr Tydfil (78) and Rhondda Cynon Taf (71), also have higher PeCa/PeIN incidence (5.96, 3.68 and 3.39, respectively), suggesting that greater deprivation is associated with increased incidence (Table 1 and Figure 4). This pattern is largely concentrated in the Valleys of Southeast Wales, historically among the most deprived regions. Conversely, less deprived areas such as Powys (24) and Monmouthshire (20) generally report lower incidence, although exceptions exist (e.g., Monmouthshire, 2.90), indicating that additional factors may also influence outcomes. Overall, these findings align with evidence that higher socioeconomic deprivation correlates with higher rates of PeCa/PeIN. The underlying mechanisms are likely multifactorial, potentially including reduced health literacy, barriers to healthcare access, poor hygiene and differences in preventative behaviours. Addressing these inequalities is therefore essential for effective prevention and early detection strategies.

### 4.7 Prevention strategies

The primary goal of this incidence report is to identify the disease burden across local authorities to inform regional public health strategies. Early diagnosis remains crucial, as outcomes decline sharply with nodal involvement.^3^ Five‐year survival exceeds 90% for localised penile cancer but falls significantly with advancing nodal metastatic involvement (75% for N1, 60% for N2 and 35% for N3 disease, respectively).^3^, ^15^, ^16^ These figures support the prognostic significance of early detection and prompt referral.

Since, PeCa/PeIN is rare and typically presents as an overt cutaneous lesion, population‐based screening programmes are neither practical nor cost‐effective. Instead, public health interventions should focus on increasing awareness among patients and clinicians to encourage early presentation and 2‐week‐wait referral for suspicious lesions.^10^

Preventive strategies targeting modifiable risk factors are also essential. Smoking cessation initiatives and HPV vaccination programmes, now universally offered to adolescent boys in the United Kingdom, represent the most effective population level measures for reducing disease burden.^14^, ^17^ In regions where the risk factors are more complex and perhaps socio‐political, such as deprivation, health inequality and life expectancy, a patient and clinician educational approach must be encouraged to improve awareness, presentation, early referral and prompt treatment.

## 5 LIMITATIONS

This study presents 15‐year average incidence rates of PeCa/PeIN but does not provide a detailed epidemiological analysis. Data on the annual number of new cases within each local authority and patients' ages at diagnosis were not available, preventing calculation of age‐standardised incidence rates (ASIR) or assessment of temporal trends. Additionally, demographic variables such as co‐morbidities and individual risk factors were not captured. These omissions represent important limitations and mean that the findings only offer an overview of PeCa/PeIN incidence in Southeast Wales.

Despite these limitations, the data used are considered robust. All cases were sourced from the PeCa/PeIN MDT database and operative records at the Royal Glamorgan Hospital, the tertiary referral centre for Southeast Wales, with data collection conducted at the time of diagnosis by the study team. Only the outward portion of the patients' postcodes (e.g., ‘CF21’ rather than the full post code ‘CF21 0DC’) was recorded. While this protects patient confidentiality, it may have introduced minor coding inaccuracies. Some postcode areas span more than one local authority, potentially resulting in a small number of mis‐assigned cases. Although this could have slightly affected local authority‐specific incidence figures, the overall incidence rate of 2.61 per 100 000 males is likely to remain accurate.

The interpretation of regional ‘hotspots’ should also be approached with caution. PeCa/PeIN is a rare disease, and small variations in case numbers can lead to substantial fluctuations in calculated incidence rates. Nonetheless, as this dataset represents a 15‐year average, the consistent clustering observed around the Valleys and urban areas of Southeast Wales suggests genuine patterns of increased incidence that merit further investigation. Mapping these areas may help inform targeted public health strategies to promote earlier detection and ultimately reduce disease morbidity and mortality.

Population denominators were derived from mid‐year male population estimates provided by ONS and StatsWales.gov, which represent the most reliable and standardised source of population data available. Although such estimates are subject to minor migration‐related fluctuations, the effect on incidence rates is likely to be negligible.

Lower incidence rates observed in regions such as Powys may reflect differences in healthcare access or patient referral patterns, with some individuals potentially seeking care outside their local health board. However, this is unlikely to have a significant impact, as all PeCa/PeIN referrals in this region are directed to the Royal Glamorgan Hospital, the designated tertiary centre for Southeast Wales.

Finally, a methodological limitation of this study is the comparison of a 15‐year average incidence rate (2009–2024) with single‐year annual incidences. Ideally, comparisons should be made using equivalent timeframes to ensure methodological consistency and strengthen the validity of inter‐study comparisons.

## 6 CONCLUSION

This study presents the first regional analysis of PeCa/PeIN incidence in Southeast Wales over a 15‐year period. The incidence rate of 2.61 per 100 000 males is notably higher than previously reported UK and global figures, suggesting a greater disease burden in this region. Distinct regional variation was observed, with the highest rates in Blaenau Gwent, Merthyr Tydfil and Rhondda Cynon Taf—areas characterised by high socioeconomic deprivation and smoking prevalence.

These findings highlight the strong association between deprivation and increased PeCa/PeIN incidence. Targeted public health strategies focusing on modifiable risk factors, particularly smoking cessation, HPV vaccination, and improved awareness of early symptoms, may help reduce incidence and improve outcomes. While population screening is not feasible, emphasis should be placed on education, early presentation and timely referral. This study provides a foundation for future research into temporal trends and risk‐adjusted regional incidence in Wales.

## AUTHOR CONTRIBUTIONS

**Joseph Latham:** Conceptualisation; methodology; validation; formal analysis; investigation; data curation; visualization; writing—original draft; writing—review and editing. **Oliver Lane:** Validation; formal analysis; visualization; writing—review and editing. **David Gower:** Validation; formal analysis; visualization; writing—review and editing. **Adrian Sale:** Methodology; investigation; writing—original draft. **Anthony Shanahan:** Conceptualisation; data curation; writing—review and editing. **Mohammed Ali:** Writing—review and editing; supervision. **Gareth Brown:** Conceptualisation; formal analysis; writing—review and editing; supervision.

## CONFLICT OF INTEREST STATEMENT

The authors declare that no conflicts of interest exist.
